# Advancing patient engagement: youth and family participation in health research communities of practice

**DOI:** 10.1186/s40900-018-0094-2

**Published:** 2018-03-12

**Authors:** Roberta L. Woodgate, Melanie Zurba, Pauline Tennent

**Affiliations:** Rady Faculty of Health Sciences, College of Nursing, University of Manitoba, 89 Curry Place, Winnipeg, MB R3T 2N2 Canada

**Keywords:** Research communities of practice, Participation in health research, Patient engagement, Youth and families

## Abstract

**Plain English summary:**

The involvement of patients in health research has resulted in the development of more effective interventions and policies in healthcare that respond to the needs of healthcare users. This article examines how working with youth and their families as co-researchers in health research communities of practice (CoPs), rather than just as participants, can benefit all involved. Health research (CoPs) promote an environment in which co-researchers have the opportunity to do more than just participate in the data collection phase of the research process. As co-researchers, youth and their families are able to participate, learn, and contribute to knowledge and building relationships that are designed to innovate and improve healthcare systems. However, in order to ensure engagement of youth and their families in health research that they find meaningful and rewarding, three factors have been identified as important parts of the process: promoting identity, building capacity, and encouraging leadership skills.

**Abstract:**

**Background**

Patient engagement in health research is becoming more popular as it can lead to evidence for developing the most effective interventions, policy and practice recommendations. Models of patient engagement have been evolving over the past four decades including health research communities of practice (CoPs). Health research CoPs help to break down professional barriers and enhance knowledge sharing for the purpose of improving health outcomes. In this article we consider health research CoPs when youth and their families are involved.

**Main body**

As part of an ongoing research program, we identify how insights about youth and their families’ views are taken into account as well as their specific roles in health research CoPs. We have worked with youth and their families not only as participants in health research, but instead as co-researchers in health research CoPs. As co-researchers, youth and their families are able to participate, learn, and contribute to knowledge and building relationships that are designed to innovate and improve healthcare systems. Promoting and creating the space for identity, capacity building, and leadership is integral to the engagement of youth and their families in health research in a way that they consider meaningful and rewarding.

**Conclusions**

Youth and families can play stronger and more meaningful roles in health research by adopting a CoPs approach. Further examination of the internal structures and connections between youth and families as well other actors (i.e., with service providers and special knowledge holders) within emerging health research CoPs would be advantageous for developing greater understanding and best practices around engaging youth and families in health research.

## Introduction

There is growing awareness that patient engagement in health research is not only ethically important, but leads to evidence for developing the most effective interventions, policy and practice recommendations, and planning for ongoing research [1–3]. Models for patient engagement have been evolving over the past four decades, and research that is grounded within evolved forms of stakeholder participation is typically understood as research as practice. Recently, attention has been shifting towards new forms of interpretive communities known as communities of practice (CoPs) and their potential for developing greater knowledge around participation [4–7]. CoPs do not privilege research-based evidence over experience-rooted knowledge; therefore, they have the potential to become powerful “venues for bridging traditional rifts in the health sector between research and practice, and among disciplines” ([8], p.3). CoPs have been found to enhance the performance of interventions through breaking down professional, geographical and organizational barriers, enhancing knowledge sharing, and facilitating the implementation of new processes [9]. There are examples of CoPs that have been successfully assembled in healthcare settings, especially within oncology [8]. Furthermore, training on how to develop and maintain CoPs focused on improving health within different contexts is an important emerging area in the literature around patient support and participation [7]. However, it is not yet well documented how CoPs could influence the development of research for health systems and interventions for youth (i.e., children and adolescents) and their families. In this article we examine patient engagement beyond the traditional hierarchical structures for participation towards developing a greater and more functional understanding of how youth and families are involved through health research CoPs. We do so by exploring the participation of youth and families in health research CoPs created through IN•GAUGE®, an ongoing research program led and coordinated by Dr. Roberta Woodgate. Dr. Woodgate’s research engages youth, families and caregivers, service providers, researchers, and policy makers towards building insights into the lived experiences of youth with physical and mental illness.

## Background

Patient engagement has roots in several international agreements including the WHO Alma Ata Declaration (1978) in which declaration 10 states, “The people have a right and duty to participate individually and collectively in the planning and implementation of their own health care” [10]. Since the Alma Ata, this ethos has been applied with different patient populations, including children and adolescents. The most formal and largely recognized articulation of children’s rights to participate is Article 12.1 of the United Nations Convention on the Rights of the Child (UNCRC), which asserts that “States Parties shall assure to the child who is capable of forming his or her own views the right to express those views freely in all matters affecting the child, the views of the child being given due weight in accordance with the age and maturity of the child” [11].

A few models and typologies for participation run parallel with the UNCRC standards and have been applied to youth engagement in health research. Many of such models have roots in Arnstein’s “ladder of citizen participation” [12], which Hart later modified and contextualized as being relevant to young people’s participation [13]. Almost a decade later, Shier’s “pathways to participation” typology for children’s participation in decision-making significantly revised the format of the model to five levels operating as a continuum [14]. These frameworks for participation have significantly influenced how children are regarded within research communities. Stewart however, stresses that it is difficult to find a workable definition of participation, and the popularity of incoherent definitions in health research “belies fundamental uncertainties about what [participation] entails and its associated benefits” ([15], p. 124).

Towards overcoming uncertainties around hierarchical frameworks for decision-making, Turner argues that the conceptual frames provided in the growing literature around CoPs can provide a more comprehensive understanding of health research systems aiming to have an influence on practice [16]. Lave and Wenger contributed greatly to the concept of CoPs and focused on informal and situated interactions towards achieving a better understanding of learning that is grounded in practice [17]. Later, Wenger focused on the trajectories of participation, social identities, and the effects of participation within different communities; and developed a set of indicators for CoPs [4]. Wenger defines the three core components of CoPs as the *domain*, which refers to a “concern, set of problems, or passion about a topic”; the *practice*, representing the knowledge that the group shares and generates; and, the *community*, which is the set of interpersonal relationships that are the product of engaging in learning through practice [4]. Wegner, McDermott, and Snyder developed the definition of *communities* further as “Groups of people who share a concern, a set of problems, or a passion about a topic, and who deepen their knowledge and expertise in this area by interacting on an ongoing basis” ([18], p.4). When the three elements function well, CoPs become structures that can take on the responsibility of developing and sharing knowledge [18]. le May expanded the CoPs framework to account for the outcomes and benefits of CoPs in health and social care, including knowledge sharing, learning, building social relationships, innovation, and improving organizations [19]*.*

Research communities engaging in research as practice have been studied using the CoPs framework, which has been applied to a variety of contexts (e.g., incarceration, community development, health, and education) with adults and mixed-age populations. Yet, the process of engaging youth and their families in health research communities of practice CoPs is yet to be studied in a systematic way. Furthermore, major funding agencies (e.g., Canadian Institutes of Health Research) equate patient engagement with the adult model of participation and do not give special consideration to how research should be conducted with youth and their families. In the following section we identify how insights about youth and their families’ views are taken into account as well as their specific roles in health research CoPs that are created through IN•GAUGE®, a research program focussed on building knowledge through conducting research *with* youth and their families, rather than *on* them.

## Approach: Research *with* youth and their families

Turner asserts that framing health research within CoPs should first involve translating research findings into practical implications for organizations and identifying ways of developing and “communicating evidence from social science research that demonstrates its relevance to ‘real-world’ decision-making such that it has maximum impact on healthcare policy and Practice” ([16], p. 2). Towards achieving the important next step of understanding the roles and outcomes of youth involvement in health research CoPs it is essential to hear from the youth themselves. IN•GAUGE® creates health research CoPs through the implementation of participatory research agendas with co-researchers (we use this term instead of “participants” towards acknowledging the contributions made as well as the power that has been divested according to participatory action research principles). These health research CoPs emerged through both intentional planning as well as the organic development of a web of relationships that results from sustained engagement in an area of research, depending on the particular study. Through IN•GAUGE®, qualitative and arts-based methods (such as photovoice, computerized drawings, body mapping) are applied to help amplify the voices of youth and their families, as well as explore and share research findings from the youth- and family-centred health research program through accessible strategies (such as the development of films, websites and choreography). These strategies all worked to highlight the voices of young people and families with lived experience and to ensure that the best available evidence flowing from the research is in the hands of those who influence children’s health – parents, families, healthcare professionals, decisions makers and children and youth. In considering CoPs framework in relation to IN•GAUGE®, the health and well-being of youth with physical and mental illness and their families is the *domain*. The *practice* is the development of strategies for improving child and family health and well-being, and the *community* are the youth and families, service providers, researchers, and policy makers that are brought together into action through the common concern (i.e., the research question). Learning is promoted in IN•GAUGE® health research CoPs through participatory action research protocols involving knowledge brokering and various feedback cycles with youth and families, researchers, service providers, and special knowledge holders [6, 20]. For learning to happen within health research CoPs, individuals need to be willing to contribute to the evolution of collective learning through sharing information, developing, and implementing strategies and conducting evaluations [21, 22].

## Promoting and creating the space for identity, capacity building, leadership, knowledge building, and relationship building

### Identity

**Fig. 1:**
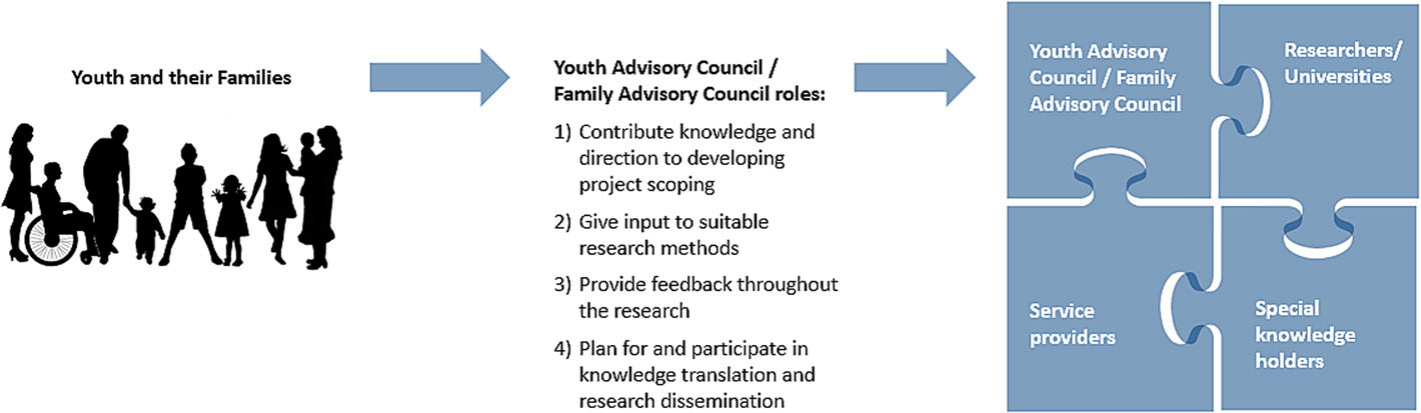
Youth and families play stronger and more meaningful roles in health research CoPs (left) through Youth and Family Advisory Councils (YACs/FACs), which in turn makes health research CoPs more connected and robust (right)

Youth and families find a sense of their roles and identities within health research CoPs that have been created through IN•GAUGE®. Co-researchers have revealed that they have learned a lot about their own journey with illness through being engaged and finding a space for reflection in the research process. Many co-researchers have reflected on how they felt at ease in the research process and viewed their participation as an opportunity to give back and help others who have similar challenges. Co-researchers often make strong statements about their identity and the ways in which their health condition is intertwined with their identity, and how that identity relates to the particular project. The building of shared identities relating to health research CoPs (i.e., feeling of belonging and being welcomed in the community) is also important, and creates the ability to transcend ways of communicating (i.e., disciplinary, cultural, generational, etc.), acknowledging other’s perspectives, and challenging assumptions [23]. Much of this communication is facilitated through the use of highly flexible interview schedules, as well as the creation of a safe space established as a result of the implementation of Youth Advisory Councils (YACs) and Family Advisory Councils (FACs). YACs and FACs contribute knowledge and direction to developing projects through project scoping, giving input on suitable research methods, providing feedback throughout the research, and planning for and participating in knowledge translation (KT) and dissemination (Fig. 1). Within IN•GAUGE®, for those studies that focused directly on youth experiences of health and illness, YACs facilitated the participation of young people in research outside of the direct influence of their families. FACs brought to light the lived experience of health and illness on families and also served to complement the work of the YACs for those projects more focused on youth experiences. Multi-directional communication and critical self-reflection within IN•GAUGE® health research CoPs contributes to connectivity and learning across boundaries and promotes the development of a shared identity and sense of belonging within health research CoPs, one of Wenger’s (1998) indicators of CoPs. Co-researchers from IN•GAUGE® involved in YACs and FACs frequently state that they enjoyed being engaged through the research process, and state that reflecting through the research process enables them to find different interpretations of their own or a family member’s illness and disability.

### Capacity building

Capacity building for youth and their families includes skill development and the enhancement of self-esteem and the ability to build social networks. Skill development involves refining communication and advocacy skills within health research systems (i.e., through YACs and FACs), which is important for developing meaningful engagement within health research CoPs more broadly, as well as in health systems more generally [24]. Through promoting and maintaining relational qualities within the research agendas, the researchers themselves provide many opportunities enhancing the self-esteem and abilities of co-researchers to build and extend their social networks [3]. Co-researchers often express that their opinions were being taken seriously and felt empowered to communicate with others through the research. Enhancing this ability to influence social networks is especially important for youth and families who may be disadvantaged through their experiences with illness. In developing IN•GAUGE® projects it is important to be cognizant that belonging to additionally marginalized groups (i.e., Indigenous, female, etc.) can cause enhanced jeopardy for health and social outcomes (Demas, [25]), requiring special attention to how power relationships are addressed and how social relationships are enhanced within health research CoPs. It is especially important when working with such groups that partnerships are fostered, collaboration is promoted, and that shared concerns are explored (i.e., the domain) early on [26]. It is also critical to consider that social relationships may mean something different for each group of co-researchers and can be influenced by factors such as culture and regional norms [27, 28]. For example, in an IN•GAUGE® study exploring African newcomer experiences with the Canadian health system, co-researchers developed an ability to extend their influence and knowledge that they then used to advocate for improved access to health services in the context of a change in political system (i.e., government cutbacks for newcomer health services).

### Leadership

Co-researchers’ learning about identity and the development of new skills eventually lead to greater participation and leadership in projects. A parent of a child with complex care needs talked about their long-term involvement in an IN•GAUGE® study and how being asked to give their thoughts on the research process and dissemination of research findings (i.e., through participating in a video documentary) enabled them to create meaning and engage in deep reflection and learning through the process. Through advisory councils, co-researchers take up a number of leadership roles and have demonstrated commitment and interest in the research reaching its full potential. Co-researchers also report finding spaces within health CoPs where they can directly impact their day-to-day care through finding new pathways for informing service providers about their particular needs. The leadership skills of co-researchers are brought into the initiation and development stages of the research through providing spaces for co-researchers to shape research priorities, project design and methods.

### Knowledge building

Patient engagement involves acknowledging that youth and families have certain knowledge and skills, but that they also will gain knowledge and skills through being involved in the health research CoPs [2]. Likewise the experience of knowledge sharing and building holds true to others who may be involved in research (e.g., clinicians), as well as the researchers themselves. Furthermore, direct interactions through health research CoPs are especially important for picking up on social cues and developing critical understanding of the lived experiences of youth and families [29]. Through working with co-researchers it was possible to build knowledge around the topics being researched, as well as knowledge contributing to how to develop approaches within ongoing and future health research CoPs. Co-researchers are keen to be part of the reflexive research design and are asked to give feedback regarding different stages of the research. Innovations in the research process occur through integrating the feedback and striving to find new and better ways to bring youth and families into health research CoPs as fully engaged co-researchers. Through YACs and FACs, co-researchers provide key knowledge for the analysis of data and KT. A few examples include the direct input into content and design for a KT website, involvement in the editing of a video documentary, and feedback on the artistic interpretation of research findings.

### Relationship building

Relationship building is an essential component to ethical research engaging youth and families [22], and is central to IN•GAUGE® health research CoPs [3]. Relationship building occurs among youth and families, as well as among youth, families, and different members of the IN•GAUGE® health research CoPs (Fig. 1). Social relationships that are fostered through previous interactions (i.e., through systems of care) between the different co-researchers (i.e., youth and their families, university researchers, service providers, and special knowledge holders) act as foundations for many of the IN•GAUGE® projects. Co-researchers are invited into collaborative spaces where their perspectives are heard and given full consideration and see YACs and FACs as a safe spaces to share experiences with each other and create sense of community. Building relationships in this way lead to the development of respect, knowledge, awareness, and understanding of knowledge within the community and enable youth and their families to contribute in more meaningful ways to health research CoPs. It is also important to acknowledge that being involved in health research CoPs can be burdensome for co-researchers and that the risks associated with tokenistic participation could be managed in part by creating measures to equally value the commitments of co-researchers, such as through adequate remuneration (depending on context, specific research project and contributions, time commitments, etc.). Co-researchers that are part of IN•GAUGE® health research CoPs are given honorariums, as well as other types of compensation (e.g., meals and transportation). Such protocols are put into place to demonstrate respect, value and commitment towards co-researchers. In some situations it is appropriate for co-researchers to be employed as paid staff members on a project as a way of formalizing their roles and compensating them for their knowledge, experience and contributions. Co-researchers involved in IN•GAUGE® YACs and FACs are also often given the option being a co-investigator or consultant to projects. Such categories came with different benefits and types of payments.

## Limitations

This interpretation mainly focused on the engagement of youth and families within health research CoPs through exploring their interactions with university researchers. This paper would have been strengthened by working with co-researchers in its development however time constraints for co-researchers and prior commitments to other knowledge translation activities by members of YACs made such an approach challenging, reflecting broader challenges of working in a participatory manner. Further examination of the internal structures and connections between the other actors (i.e., with service providers and special knowledge holders) within emerging health research CoPs would be advantageous for developing greater understanding and best practices around how health research CoPs function as entire systems [30]. Further investigations into the structures of YACs and FACs would also be beneficial for understanding their impacts of health policy and practice [31].

## Conclusions

The dearth of health literature focusing on patient engagement involves frameworks equating patients with adults, not recognizing that these approaches may not resonate with youth. Through exploring the outcomes of engaging youth and their families through IN•GAUGE®, a sixteen yearlong research program led by Dr. Roberta Woodgate that is focused on working with youth and their families across the spectrum of patient engagement, improving health research and practice, we have gleaned several important insights about the development of health research CoPs for health systems for youth and their families. Promoting and creating the space for identity, capacity building, and leadership is integral to the meaningful engagement of youth and their families in health research. Within such conscious spaces, co-researchers are able to participate, learn, build social capital, and contribute to knowledge and building relationships that are designed to innovate and improve healthcare systems.

## Abbreviations

CoPs: Communities of Practice
FAC: Family Advisory Council
UNCRC: United Nations Convention on the Rights of the Child
WHO: World Health Organization
YAC: Youth Advisory Council
